# *Opinion*: Why Sex‐Based Genomic Differentiation Should Not Be Overlooked in Population Genetics

**DOI:** 10.1111/mec.70061

**Published:** 2025-08-07

**Authors:** Yu‐Chi Chen, Nikolas Vellnow, Justin J. S. Wilcox, Sahar Javaheri Tehrani, Toni I. Gossmann

**Affiliations:** ^1^ Computational Systems Biology, Faculty of Biochemical and Chemical Engineering TU Dortmund University Dortmund Germany

**Keywords:** evolutionary genomics, genomic variation, local adaptation, population genetics, reference genome, sex‐based differentiation, sex‐biased selection, SNP‐based analysis, W chromosome, Z chromosome

## Abstract

Sex‐specific genomic differentiation is a crucial yet frequently overlooked factor in population genetics. In this opinion piece, we leverage the substantial genomic resources available for the great tit ( *Parus major* ), including population‐scale data sets from many European populations, to investigate genomic differentiation between males and females. Unlike in some other species, where high‐quality genome assemblies exist but broad population sampling is lacking, the great tit offers a unique opportunity to study sex‐based differentiation at both the genomic and population level. We identify significant differentiation at an autosomal locus on chromosome 5, which we hypothesise originates from sex‐linked variation present on the sex chromosomes (Z and potentially W). By referencing genomic data from other songbirds with well‐assembled sex chromosomes, we illustrate how autosomal loci may exhibit high sequence similarity to sex‐linked regions. Our analyses demonstrate that uneven sex ratios in sampled populations can substantially bias differentiation metrics (e.g., *F*_*ST*_), potentially resulting in false‐positive interpretations of adaptive differentiation. To mitigate such issues, we stress the importance of sex‐aware study designs, including balanced sex sampling and explicitly incorporating sex as a covariate. Furthermore, while optimal study designs would include high‐quality reference genomes from both sexes, we recommend, as a pragmatic and cost‐effective alternative for labs with limited resources, generating a reference genome from the heterogametic sex (females in birds) to ensure both sex chromosomes are represented in mapping and analysis. Finally, we emphasise the need for rigorous validation of candidate loci to ensure accurate and biologically meaningful outcomes in evolutionary genomic studies.

## 1 Motivation

Understanding how populations adapt locally is fundamental to evolutionary biology because it reveals the genetic mechanisms that allow species to persist across heterogeneous environments (Savolainen et al. 2013; Ye et al. 2023; Kawecki and Ebert 2004). Patterns of genetic differentiation among populations often reflect adaptation to local ecological pressures—such as temperature, food availability or pathogens—which in turn can influence conservation strategies by identifying distinct management units and informing policy (Moritz 1994; Frankham et al. 2010; Jump and Peñuelas 2005). Characterising adaptive genetic variation also enables predictions about how biodiversity may respond to rapid environmental changes, including climate change, habitat fragmentation and other anthropogenic impacts (Frankham et al. 2010; Hoffmann and Sgrò 2011; Lyam et al. 2022; Ord et al. 2023; Gossmann et al. 2019; Popovic and Lowry 2020). Genome‐wide association studies and advanced population genomic modelling are increasingly used to uncover the genetic architectures underlying population‐specific traits and to understand how biodiversity is maintained (Luikart et al. 2003; Santure and Garant 2018; Hoffman et al. 2024; Gossmann et al. 2010; Romiguier et al. 2014; Burri et al. 2015; Ellegren and Galtier 2016).

Despite major advances in the field, sex‐specific genetic differentiation remains comparatively underexplored, even though males and females frequently differ in their ecological roles, behaviour and physiology—differences that can translate into distinct evolutionary pressures and divergent genomic signatures of adaptation (Cheng and Kirkpatrick 2016; Rogers et al. 2020; Campagna and Toews 2022; van den Heuvel et al. 2023; van Oers et al. 2023; Lindner et al. 2024). For example, sexually antagonistic selection, differential demographic histories or sex‐linked inheritance can generate patterns that mimic or mask genuine signals of local adaptation (Hoban et al. 2016; Lasne et al. 2017; Ruzicka et al. 2020; Flintham et al. 2024). If not explicitly accounted for, such sex‐based effects risk producing misleading inferences, especially when standard genome‐wide scans pool individuals of both sexes or when sex ratios are unbalanced in sampling (Li and Merilä 2010; Cheng et al. 2021; He et al. 2022; Gong et al. 2022).

A critical, yet often overlooked, issue is the potential for sex‐ratio bias in population genomic data sets. Uneven representation of males and females can artificially inflate or deflate measures of genetic differentiation, such as *F*_*ST*_, producing false‐positive or false‐negative signals of local adaptation (Foll and Gaggiotti 2008; Yi et al. 2010). Furthermore, unbalanced sex ratios can distort site frequency spectrum statistics (e.g., Tajima's *D*), environmental association tests or haplotype‐based selection metrics if sex is not included as a covariate (Rodrigues and Dufresnes 2017; Pearman et al. 2022). Recent studies have shown that technical artefacts—such as mapping errors, structural variants or incomplete reference genomes—can sometimes produce apparent sex‐specific differentiation on autosomal regions, even in the absence of true biological sex linkage (Gong et al. 2022; Bailey et al. 2023; Makova et al. 2024).

The great tit ( *Parus major* ) provides an exceptional model for investigating these issues. Its broad distribution across Europe and Asia, extensive ecological and behavioural research history and the availability of high‐quality reference genomes and population‐scale SNP and resequencing data have positioned it at the forefront of evolutionary genomics and adaptation studies (Gossmann et al. 2014; Laine et al. 2016; Corcoran et al. 2017; Delaitre et al. 2023; Spurgin et al. 2024; Stonehouse et al. 2024). The clearly established avian ZW sex‐chromosome system and recent interest in the genetic architecture of adaptation make the great tit a powerful system to dissect the roles of sex‐specific differentiation in shaping population structure and adaptive divergence.

In this study, we analyse genomic data from multiple great tit populations across Europe to illustrate how unrecognised sex‐linked artefacts can affect estimates of population differentiation. Our goal is to demonstrate that such artefacts have the potential to bias downstream analyses in population genomics, including studies aiming to detect local adaptation. We specifically hypothesise that:

1. Sex‐biased genomic differentiation will be evident not only on the sex chromosomes (Z and W) but may also occur in certain autosomal regions as a result of duplicated sequences or sex‐linked structural variants.
2. Imbalanced sex ratios can inflate or obscure signals of population differentiation and may generate false‐positive signatures of local adaptation in genome‐wide scans. Explicitly accounting for sex—either as a covariate or through balanced sampling—will improve the accuracy of detecting genuinely adaptive loci and reduce the risk of misleading inference.

By addressing these predictions and synthesising both empirical and theoretical insights, we aim to demonstrate the necessity of a sex‐aware approach for robust inference in population genomics, and we propose practical guidelines for future studies investigating local adaptation and evolutionary processes.

## 2 Case Study: Great Tit HapMap Data

**FIGURE 1:**
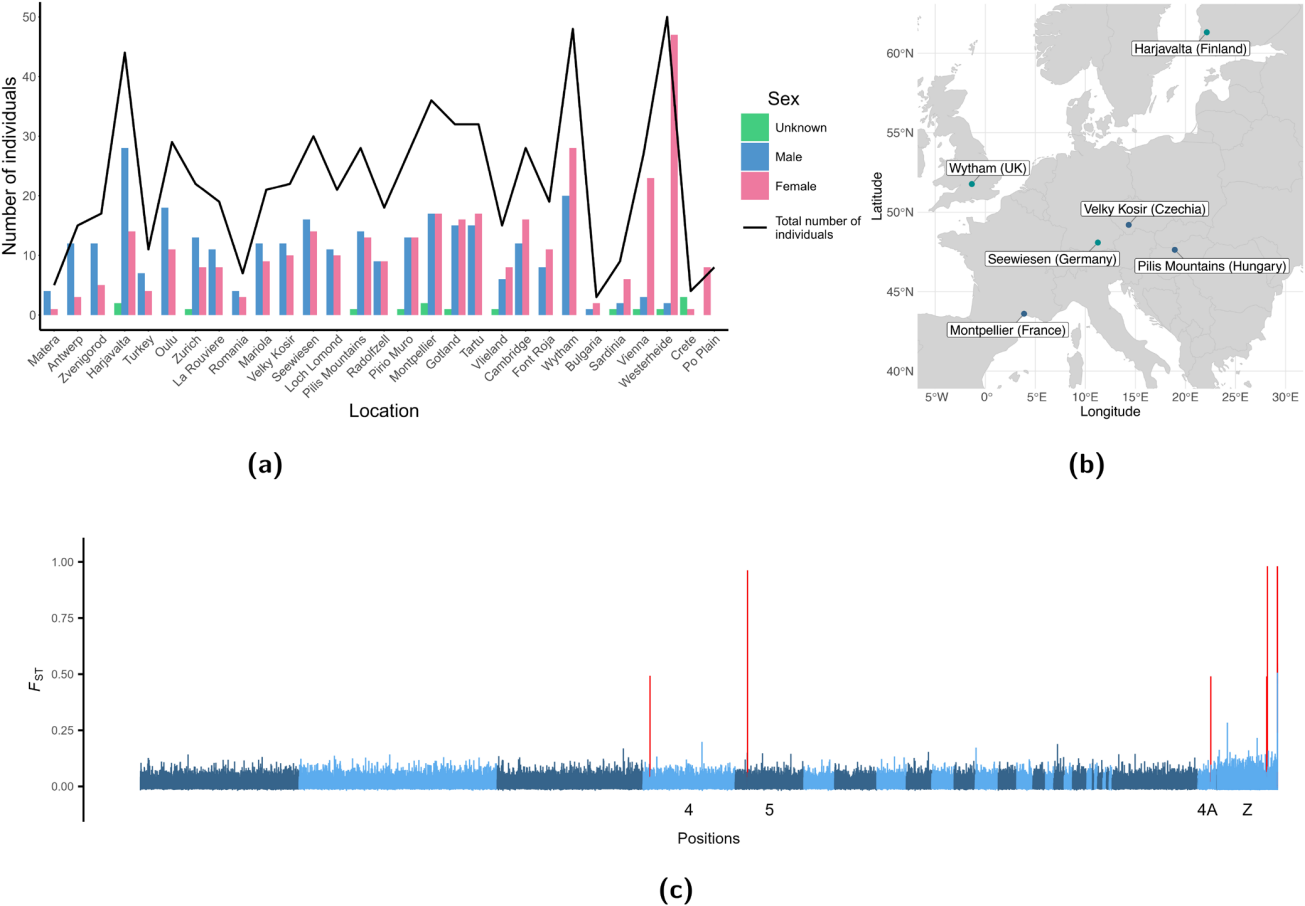
Individual sex identification, study population locations and genomic differentiation between sexes. (a) Sex identification based on SNPchip heterozygosity on the Z chromosome for 647 individuals. Shown are the number of male, female and unidentified (‘Unknown’) individuals for each population along with the total number of individuals per population. Individual inbreeding coefficients (*F*) on the sex chromosome, as computed by PLINK were used. Under a ZZ/ZW system (female is the heterogametic sex), F values cluster near 0 for males (ZZ) and near 1 for females (ZW). Note, that for some individuals, sex identification is inconclusive (Unknown, 0.2 < *F* < 0.8). (b) Locations of the six populations analysed in this study. We restricted our analysis to six populations with at least nine female and nine male individuals, respectively. Cyan and dark blue coloration denotes the two types of populations depicted in Figure 3. (c) Genomic differentiation between male and female individuals measured as *F*_*ST*_ (Weir and Cockerham 1984) of 54 male and 54 female individuals, sampled from six populations. Shown are *F*_*ST*_ values for every SNP. Several peaks with $F_{\mathrm{ST}}>0.2$ were identified (indicated in red), one on chromosome 5 and two on chromosome Z are close to one.

Here, we examine sex‐specific genomic variation across European great tit populations (Spurgin et al. 2024; Stonehouse et al. 2024), in particular in the light of the potential impact of sample composition on inferred patterns of local differentiation. We account for sex and sample differences in large‐scale population genetic samples by focusing on the great tit HapMap data that includes samples from 29 local populations across Europe (Spurgin et al. 2024; Stonehouse et al. 2024). We first identified the sex of individual birds based on heterozygosity measures of the presumed Z chromosome (Figure 1a). This approach allowed us to reliably distinguish males from females for the majority of samples (297 males and 335 females were reliably identified, while 15 remain undetermined, Table S1).

First, we observed substantial variation in sampling effort across populations, with the total number of individuals per population ranging from as few as four to more than 50 (Figure 1a). Second, the proportion of female individuals in each population was highly variable, spanning from 10% to 100%. Given this substantial heterogeneity, we ensured balanced representation of sexes in our analyses. Specifically, we (randomly) selected nine female and nine male individuals from six populations (108 individuals in total) for further analysis: Seewiesen in Germany, Wytham in the United Kingdom, Harjavalta in Finland, Montpellier in France, Pilis Mountains in Hungary and Velky Kosir in Czechia (Figure 1b).

First, using the 108 individuals from the six populations, we grouped all 54 females and 54 males together and conducted a genome‐wide differentiation scan contrasting the sexes (54 males vs. 54 females). Principally one would expect limited genetic differentiation between males and females of the same population on the autosomal chromosomes between individuals of the same population. However, this analysis revealed a strong signal of such differentiation ($F_{\mathrm{ST}}\approx 1$) across the six populations on chromosome 5 at position 10,662,578 bp (Figure 1c). Additionally, multiple sites with elevated differentiation were observed on the presumed Z chromosome as well as one on chromosome 4 and 4A (Table 1). Because genomic differentiation between the sexes at loci on the Z chromosome, or autosomal loci with *F*_*ST*_ values around 0.5, can parsimoniously be explained by the presence of a W‐linked homologue in females (as outlined in Table S2), we excluded such hits from further consideration. Instead, we focused our analysis on SNPs with *F*_*ST*_ values approaching 1 on autosome 5, as such extreme differentiation is not easily attributable to simple dosage differences between Z and W.

**TABLE 1:** Summary of genomic regions with elevated sex‐specific differentiation.

Chromosome	Position(s) (bp)	*F*_*ST*_ range	Number of SNPs
4	5,481,317	0.49	1
A	12,433,942	0.49	1
5	10,662,578	**0.96**	1
Z	64,160,871; 74,482,096	**0.98**	2
Z	57,542,385; 74,462,828–74,512,700	0.41–0.55	12

*Note:* Listed are genomic regions with single SNP *F*_*ST*_ > 0.4 between 54 males and 54 females across six great tit populations. Z chromosome hits are grouped by level of differentiation, *F*_*ST*_ values close to one are displayed in bold. Coordinates refer to genome assembly 1.04 of *Parus major* . Loci of differentiation are likely caused by assembly and mapping artefacts.

## 3 Case Study: Locus of Interest on Chromosome 5

**FIGURE 2:**
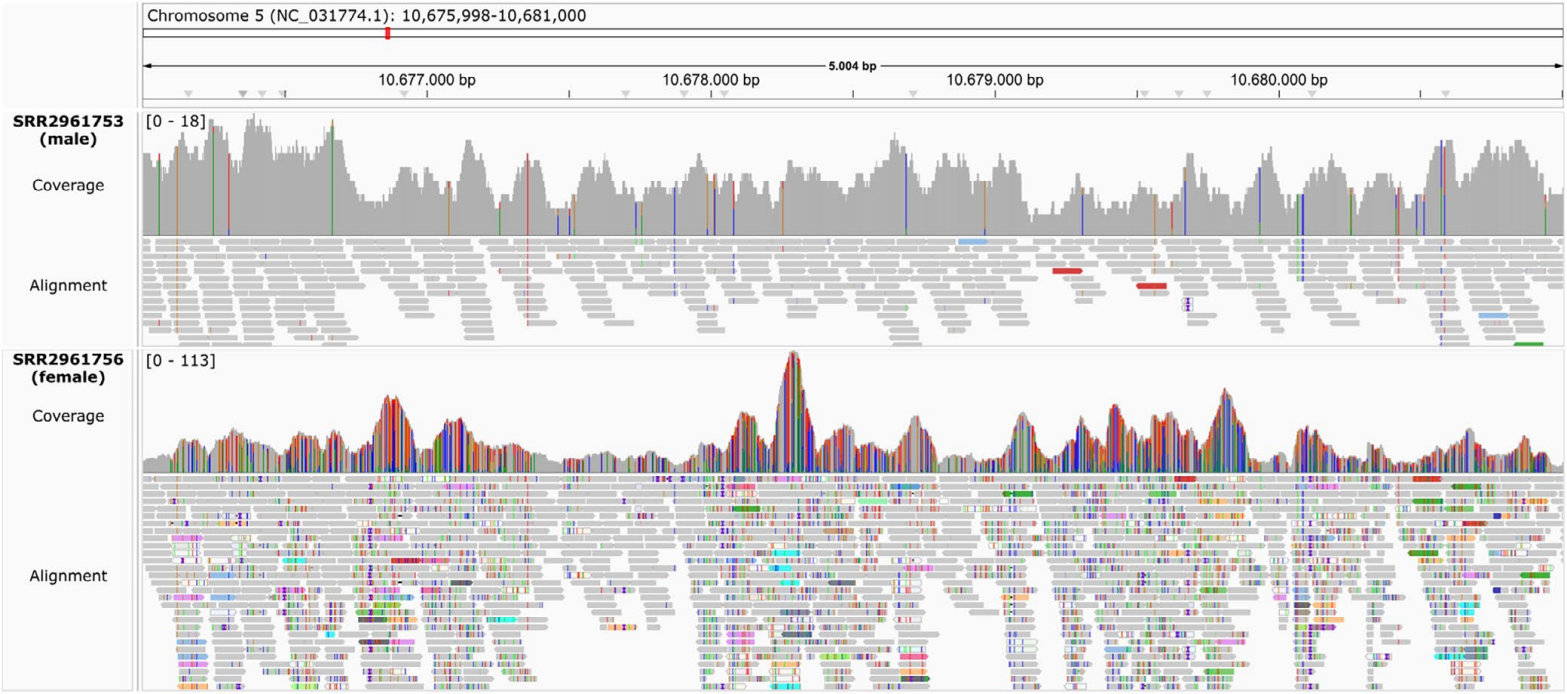
Detailed comparison of whole genome sequencing read mappings between male and female great tits in the most differentiated region on chromosome 5. Shown are histograms of sequencing coverage and the aligned Illumina reads of a male (SRR2961753, top) and a female (SRR2961756, bottom) individual from Wytham, United Kingdom. Sites that vary from the reference are marked in colour according to the default settings of the Integrated Genomics Viewer. Note the higher coverage and nucleotide differentiation in the female.

To further investigate the observed pattern of genomic differentiation on chromosome 5, we inspected mapped reads (from whole‐genome sequencing) of this region in one male and one female bird from Wytham woods, United Kingdom (other individuals show very similar patterns, not shown). We found an up to sixfold higher coverage as well as a higher differentiation from the reference in the female compared to the male (Figure 2). This suggests that the pattern found in the genome‐wide differentiation scan might be driven by a region of several kb and not just one SNP.

**FIGURE 3:**
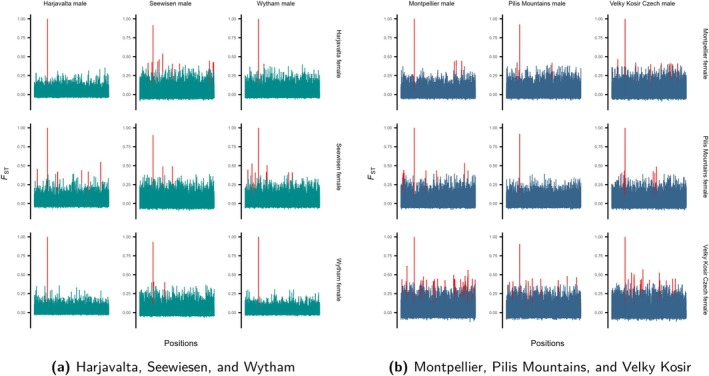
Population‐specific patterns of genetic differentiation between females and males on chromosome 5. Shown are *F*_*ST*_ values for every SNP based on a comparison between female populations versus male populations where an *F*_*ST*_ peak close to one at position 10.66 MB is visible. (a) Harjavalta, Seewiesen and Wytham and (b) Montpellier, Pilis mountains and Velky Kosir. Several elevated *F*_*ST*_ > 0.4 peaks can be observed as well, indicated in red.

We also investigated sex‐specific genomic differentiation in local populations specific to chromosome 5. We find that the observed global differentiation is visible in the local populations as well: Differentiation between male and female individuals was very high at the locus on chromosome 5 (Figure 3a,b, $F_{\mathrm{ST}}\approx 1$ at position 10,662,578 bp on chromosome 5). We also see several elevated $F_{\mathrm{ST}}>0.4$ when contrasting males and females between and within local populations which is not necessarily symmetric (e.g., Montpellier male vs. Czechia female in contrast to Czechia male vs. Montpellier female, Figure 3b). Although these findings may indicate sex‐specific local differentiation, they could also be driven by the relatively small sample size used in this study. With nine individuals per sex, individual SNPs may appear to have inflated $F_{\mathrm{ST}}$ values due to statistical noise (Nazareno et al. 2017). Consequently, increasing the sampling effort per population would be a valuable next step to verify these results.

BLAST searching (Camacho et al. 2009) of the region flanking this site against other passerines from GenBank (Benson et al. 2012) provides clear evidence that there is a W‐linked homologue of this region (Figure S1). In fact, what was thought to be an SNP is actually a single nucleotide difference between the autosomal and W‐linked homologues, such that females appear to have two ‘alleles’ and males just one, leading to genotypes that are fixed within sex but different between the sexes (Jon Slate pers. Comm.). This finding is also consistent with the higher nucleotide differentiation in the female compared to the male found in the mapped whole‐genome sequencing data (Figure 2). To further investigate this, we used reparameterised BLAST runs to align the SNP‐chip probe for the locus on chromosome 5 specifically against the *Poecile atricapillus* genome, which features chromosome‐scale assemblies for both the Z and W chromosomes. The probe produced multiple hits on the Z and W chromosomes, as well as on autosomes 32 and 26 (Figure S2, Table 2). The best‐scored alignment showed three mismatches on the W chromosome with an identity of 98.6%. These findings illustrate that the observed genomic differentiation is not exclusive to great tits but instead exemplifies a broader pattern of sex‐specific genetic differentiation prevalent across various passerine bird species. This pattern arises due to differences between autosomal and sex‐linked homologues, rather than true polymorphic variation of a single autosomal locus.

**TABLE 2:** Summary of BLAST hits of the chromosome 5 probe sequence against the *Poecile atricapillus* genome assembly.

Chromosome	Type	Number of hits	Percent identity range (%)
W (NC_081288.1)	W sex chromosome	7	98.4–98.6
Z (NC_081289.1)	Z sex chromosome	19	96.9–97.4
32 (NC_081280.1)	Autosomal (32)	6	95.5–96.1
26 (NC_081274.1)	Autosomal (26)	7	94.0–94.7

*Note:* Percent identity ranges and number of hits per chromosome are listed.

## 4 Case Study: Implications for Population Genetic Differentiation

**FIGURE 4:**
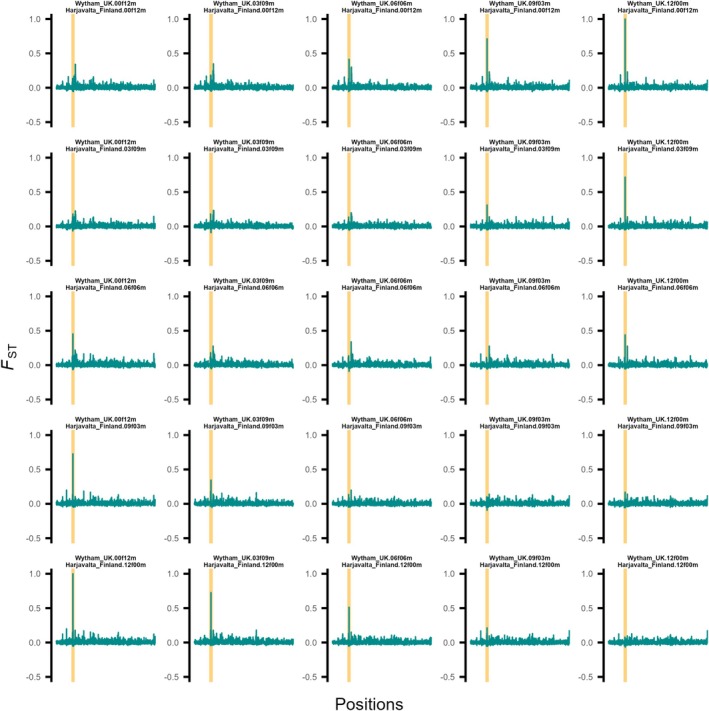
Population differentiation index (*F*_*ST*_) on chromosome 5 for Harjavalta versus Whytham population. Shown are *F*_*ST*_ values for every SNP. For all plots, the same two populations with 12 individuals each are contrasted but varying numbers of male and female individuals are used. Note that the number of females and males for each contrast is denoted as two digits followed by female (f) or male (m). For example, WythamUK.03f09m would denote three female and nine male individuals from the Wytham population. Note, the extent of the *F*_*ST*_ peak at the locus on chromosome 5 (highlighted in yellow) varies across the different contrasts depending on the number of females and males in both populations.

To explore the potential impact of sex ratio on genomic differentiation at the identified locus on chromosome 5, we conducted subsampling with varying proportions of male and female individuals. Specifically, we tested the Harjavalta population (Finland) versus the Wytham population (UK) which both had at least 12 male and female individuals. This allowed us to use different sex ratios of 0:12, 3:9, 6:6, 9:3 and 12:0 to measure genomic differentiation between the same population with varying number of males and females (Figure 4). We find that *F*_*ST*_ values could range from 0 to 1, depending on the sex ratio in the sample, for both populations. In particular, for the case when one of two population samples would consist of single sex individuals only, we would observe that the strength of differentiation would depend on the sex ratio of the contrasted population (Figure 4, first and last column and row, respectively).

## 5 Discussion

Sex‐specific genomic variation likely contributes to local adaptation by reflecting divergent evolutionary pressures between males and females. Traits advantageous to one sex in a particular environment may conflict with those beneficial to the other, resulting in intralocus sexual conflict and driving sex‐biased selection. However, identifying the genomic basis of female/male differences remains challenging, particularly when distinguishing true sex‐specific genetic effects from technical or biological sources of background variation. In this study, we aim to highlight these difficulties by analysing whole‐genome sequencing data from both sexes. Using a subset of data from the great tit HapMap project, we identified 17 SNPs showing strong differentiation between 54 males and 54 females. This illustrates both the potential for, and the challenges inherent in, detecting sex‐specific genomic differences.

Three of 17 sex‐specific SNPs were located on autosomes. These may represent technical artefacts, potentially arising from the absence of the W chromosome in the reference genome used when the SNP chip was designed, or from repetitive elements or translocations present in only one sex. While whole‐genome resequencing identified broader regions of sex‐specific differentiation (Figure 2), single SNP analyses, as presented here, often lack the resolution to capture consistent patterns across larger genomic regions. Extreme differentiation observed at individual SNPs is unlikely to persist throughout entire loci, suggesting that previous reports of differentiation (Spurgin et al. 2024; Stonehouse et al. 2024) are probably not significantly impacted by these findings. Additionally, our study lacked W chromosome data, preventing direct analysis of female‐specific regions.

Our analyses identified strong sex‐specific differentiation at a locus on chromosome 5, which initially suggested the possibility of autosomal sex‐linked differentiation. However, closer inspection indicated that this pattern was more likely due to a combination of genome assembly and mapping artefacts. Such artefacts can arise either from misassembly, where fragments from sex chromosomes are incorrectly assigned to autosomes or from genuine biological events such as the translocation or duplication of repetitive elements between sex chromosomes and autosomes. In the case of a simple misassembly of a Z or W fragment onto an autosome, we would expect females, who possess only one Z chromosome, to have lower sequencing coverage than males at affected loci, and males to show heterozygosity, leading to moderate differentiation (*F*_*ST*_ around 0.5). In contrast, our results showed higher sequencing coverage in females and near‐complete differentiation (*F*_*ST*_ approaching 1), suggesting a more complex underlying mechanism that may involve both technical and biological sources.

Further analysis revealed extensive homology of this region to sequences on both sex chromosomes (W and Z) and to multiple autosomal regions in related songbird species. This pattern strongly supports the interpretation that repeated elements or paralogous regions, whether due to assembly artefacts or genuine copy number variation, may underlie these observations. The lack of a fully assembled W chromosome in the great tit reference genome likely means that reads from W‐specific sequences are mis‐mapped to homologous regions on autosomes or the Z chromosome. Such widespread repetitive sequences and the resulting mapping ambiguities represent a significant potential source of error in genomic analyses. Therefore, apparent autosomal sex‐specific differentiation observed in genomic studies, particularly in non‐model organisms without comprehensive reference assemblies, should be interpreted with caution. These signals may result from either technical artefacts, such as genome misassembly, or true structural variation, such as translocations or duplications, rather than genuine biological differences between the sexes.

**FIGURE 5:**
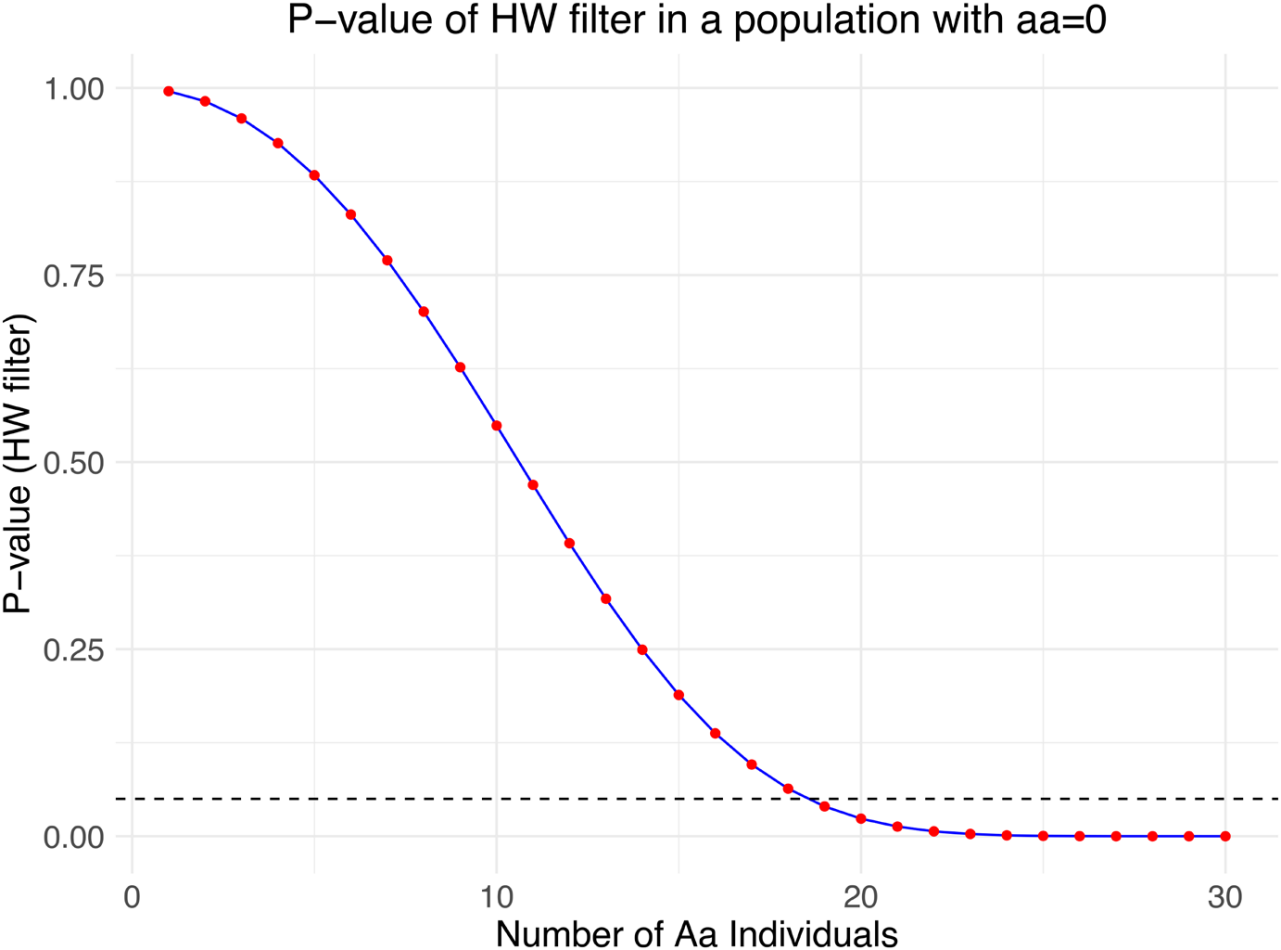
*p* Value of $\chi^{2}$ test for Hardy–Weinberg‐filtering of a population where only AA and Aa individuals are observed. This could be relevant when males result in AA genotype calls and females in Aa genotype calls. In such a case, at least 19 females of 30 individuals would be necessary to obtain a significant deviation from Hardy–Weinberg equilibrium. Dashed horizontal line indicates the 5% significance level.

Sample size plays a crucial role in population genomic analyses. SNP chips offer a cost‐effective way to genotype a large number of individuals, enabling the application of Hardy–Weinberg (HW) filters. However, if local populations exhibit unique genomic abnormalities, separate HW filtering may be required for each subpopulation which has its own caveats (Pearman et al. 2022). Our study identified two distinct types of sex‐specific SNPs: those with $F_{\mathrm{ST}}\approx 1$, where males and females are both homozygous for different alleles, and those with $F_{\mathrm{ST}}\approx 0.5$, where one sex is homozygous and the other sex are called heterozygous. Detecting the latter type requires sufficient sampling of the heterozygous individuals. For instance, in a sample of 30 individuals, at least 19 heterozygous individuals would be needed to reject HW equilibrium for a given SNP at the 5% significance level, potentially more when accounting for multiple testing (Figure 5). Therefore, it seems desirable to include sex information when filtering HW disequilibrium.

In addition to *F*_*ST*_, unbalanced sex ratios can also bias other commonly used metrics for detecting selection. Site frequency spectrum (SFS)‐based statistics, such as Tajima's *D* and Fay and Wu's *H*, rely on the assumption that each sampled chromosome represents an independent and random draw from the population. If one sex is underrepresented in the sample, the SFS becomes distorted. For example, when only a few individuals of one sex are sampled, there will be an excess of rare variants in the combined data set, which leads to lower (more negative) Tajima's *D* values. In contrast, more balanced sex ratios result in a higher proportion of intermediate frequency variants, which leads to higher (less negative or even positive) Tajima's *D* values. These distortions can mimic the genomic signatures typically attributed to directional or balancing selection: Methods that scan for selective sweeps using the SFS (such as SweepFinder or SweeD) may incorrectly infer recent selective sweeps when sex ratios are skewed, or detect apparent balancing selection under more balanced sampling, purely as a consequence of sampling bias rather than true evolutionary processes. Haplotype‐based statistics, such as iHS and XP‐EHH, are also vulnerable to sex‐specific artefacts. These methods measure the decay of extended haplotype homozygosity, and their accuracy depends on correctly phased haplotypes and unbiased representation of chromosomes from both sexes. If females possess additional copies of W‐linked regions (as may occur in regions of mapping ambiguity, e.g., on chromosome 5), haplotype lengths can be artificially extended, increasing the rate of false positives unless males and females are analysed separately.

Moreover, environmental association methods, including LFMM and RDA, can yield misleading results if sex ratios covary with environmental gradients, as genotype–environment associations may actually reflect underlying sex differences rather than true adaptation. Outlier detection approaches, such as BayeScan and pcadapt, also assume random mating and balanced sampling; deviations from these assumptions, such as sex imbalance, inflate both type I and type II errors unless sex is included as a covariate or analyses are conducted separately by sex.

Given these potential biases, we recommend that studies explicitly account for sex in their design and analysis. This includes maintaining balanced male‐to‐female sampling, incorporating sex as a fixed effect in statistical models, or conducting sex‐specific analyses to ensure robust and unbiased inference of selection and local adaptation.

To date, no study in great tit genomics has explicitly contrasted males versus females to search for fine‐scale, autosomal sex‐specific differentiation. Large‐scale genomic surveys (Spurgin et al. 2024; Stonehouse et al. 2024) and population‐level scans for local adaptation (Gossmann et al. 2014; Corcoran et al. 2017) have focused on allele frequency differences among populations and often excluded sex chromosomes altogether. Reduced representation studies, for example, RADseq (Huang and Rabosky 2015), and sliding window *F*_*ST*_ analyses (Delmore et al. 2023) emphasise broad genomic trends and may lack resolution to detect narrow, sex‐specific loci. Furthermore, when males and females are balanced in the same sample, any sex‐only differentiation is diluted unless specifically tested. These methodological factors may explain why the autosomal locus on chromosome 5 has remained undetected in earlier work.

The complexity of sex‐linked differentiation in genomic data sets requires not only analytical vigilance but also thoughtful methodological planning. As summarised in Table S2, even a single SNP can exhibit sex‐specific allele patterns due to a range of underlying genomic configurations—including pseudoautosomal inheritance, hemizygosity, sex‐limited duplications or structural variants on the W chromosome. These diverse scenarios can generate misleading signals of differentiation if not properly accounted for, particularly in species with ZW sex determination such as birds. Importantly, many of these patterns are not readily detectable with standard short‐read sequencing or reduced representation approaches, and their interpretation often depends on the resolution and completeness of the reference genome used. Recognising these potential configurations is therefore critical for designing robust studies of local adaptation and avoiding false‐positive signals arising from sex‐biased genomic architecture.

To mitigate the confounding effects of sex ratio imbalance in genome‐wide analyses, we recommend adopting fully sex‐aware designs. First, all selection and association analyses should, if possible, be conducted separately for males and females. This approach helps preserve informative loci and avoids the loss of data that can result from indiscriminate filtering based on sex‐linked variation. Second, sex should be explicitly modelled as a covariate in genotype–environment association and outlier‐detection frameworks, so that allele‐frequency differences due to sampling bias are accounted for rather than mistaken for adaptive signals. Third, candidate regions identified in mixed‐sex analyses should be validated with targeted molecular assays—such as digital droplet PCR, qPCR for copy number variation or long‐range amplicon sequencing—to verify whether observed signals reflect genuine sex‐linked structural variants or potential mapping artefacts.

Furthermore, we emphasise the importance of sex‐aware reference genomes. We recommend local reference genomes (Thorburn et al. 2023), both female‐ and male‐derived, to improve the resolution of sex‐specific variation. Where such references are unavailable, we recommend using the heterogametic sex (e.g., female in birds) as the reference and conducting analyses separately by sex to minimise mapping bias and misinterpretation of sex‐linked differentiation. Lastly, we strongly encourage efforts to assemble even draft versions of W chromosomes in key avian systems; mapping reads against a W‐inclusive genome can help disentangle autosomal signals from unrecognised sex‐linked paralogues. Together, these strategies can help preserve statistical power, avoid false positives and ensure more robust and biologically meaningful inference in studies of local adaptation.

While a sex‐aware study design is ideal, methodological choices are often constrained by financial and logistical realities. Different sequencing and genotyping approaches vary considerably in their capacity (Hoban et al. 2016) to capture sex‐specific genomic signals and reliably distinguish between male and female individuals (Table 3). Reduced representation methods like RAD sequencing are widely used due to their low cost and scalability. However, they often provide sparse genomic coverage and may lack sufficient markers on sex chromosomes to consistently detect sex‐linked differences or accurately infer individual sex, especially in species with homomorphic sex chromosomes or complex sex determination systems.

**TABLE 3:** Comparison of sequencing and genotyping methods for sex‐specific genomic analyses.

Method	Advantages	Disadvantages
RAD sequencing	Very low cost and scalable to large sample sizes; suitable for non‐model organisms	Reduced representation covers only a fraction of the genome; may miss rare or localised sex‐linked variation
SNP microarray (SNP chip)	Cost‐effective for genotyping large cohorts on the same marker set	Predefined marker panel may omit sex chromosome loci, introducing sex bias
Whole‐genome sequencing (short reads)	Genome‐wide discovery of SNPs and small indels across autosomes and sex chromosomes	Higher per‐sample cost; limited ability to detect large structural variants; potential bias from using a male‐derived or female‐derived reference
Long‐read sequencing	Enables de novo assembly of sex‐specific reference genomes and accurate detection of structural variation	Relatively expensive; higher raw error rates; substantial computational and storage requirements

SNP microarrays offer efficient genotyping at scale, but they depend on predefined marker panels that may omit or underrepresent sex‐linked loci. This can lead to systematic sex bias in downstream analyses and limits their utility in reliably assigning sex without prior knowledge. Whole‐genome sequencing using short reads offers much broader genomic coverage and can generally distinguish males and females if sex chromosomes are well represented in the reference genome. However, whole‐genome sequencing is still susceptible to sex‐specific reference bias: For example, mapping female reads to a male‐derived genome may obscure W‐linked sequences entirely.

Long‐read sequencing currently offers the best opportunity to resolve complex sex‐specific structural variants, detect W‐ or Y‐linked regions and generate high‐quality sex‐specific reference genomes, which are crucial for accurate sex identification and analysis of sex‐biased differentiation. In addition, long‐read technologies are particularly effective at resolving repetitive elements, large insertions and deletions and chromosomal translocations, all of which are often missed or misassembled with short‐read data (Chen et al. 2025). Despite their higher cost and computational demand, even a small number of long‐read samples, such as one male and one female, can substantially improve downstream analyses when used to anchor sex‐aware mapping strategies and enhance the detection of these important genomic features.

Ultimately, all methods have value when their limitations are accounted for. Even with reduced‐resolution methods, sex‐specific differentiation can be meaningfully analysed if samples are balanced and data are interpreted cautiously. Where possible, incorporating basic checks (e.g., sex‐specific coverage profiles or k‐mer‐based classification) can help identify sex‐linked markers and verify the sex of individuals. Especially in systems lacking annotated genomes, we recommend using the heterogametic sex as a reference, or performing separate analyses for males and females, to minimise bias and increase robustness of inference.

Simulation‐based approaches could provide critical insights into the extent and mechanisms by which sex chromosome‐related artefacts confound analyses of genomic differentiation. For example, forward genetic simulations (Haller and Messer 2023) can model populations with realistic genome architectures, including autosomes, Z chromosomes and W chromosomes, under various demographic and selective scenarios. By explicitly simulating both true sex‐linked loci and autosomal loci, as well as potential mapping or assembly artefacts such as the translocation or duplication of W‐linked sequences onto autosomes, researchers can systematically assess how different sampling schemes, such as balanced versus unbalanced sex ratios, and reference genome completeness, for example, with or without the W chromosome, affect the detection of population differentiation and signatures of selection. Such simulations would also allow for the evaluation of mitigation strategies, for example, by incorporating sex as a covariate or by using more complete reference genomes, and would quantify the likelihood of false positives under varying conditions. These approaches offer a promising framework to better understand and ultimately control for the confounding effects of sex‐specific genomic features in future studies of adaptation and population structure.

## 6 Conclusion

In this study, we uncovered sex‐ and population‐specific genomic differentiation on chromosome 5 across six European great tit populations. Our findings show that unbalanced sex ratios in population samples can significantly distort genomic differentiation metrics, such as *F*_*ST*_, potentially leading to false signals of local adaptation. We also highlight the importance of addressing technical artefacts, including misassembled regions and missing sex‐linked sequences in reference genomes. BLAST searches against the *P. atricapillus* genome revealed over 30 homologous regions for our focal locus, demonstrating that such mapping artefacts are likely widespread in passerines and may not be restricted to the single example we discuss in detail. To improve accuracy in population genomic studies, we recommend adopting sex‐aware analytical approaches, ensuring balanced sampling, enhancing reference genome completeness—particularly for female‐specific regions—and validating candidate loci rigorously. Accounting for sex‐based genetic variation is essential for drawing reliable and biologically meaningful conclusions in studies of adaptation and evolutionary genomics.

## 7 Materials and Methods

### 7.1 Data Sources

We utilised a publicly available genomic data set from the great tit HapMap project (Stonehouse et al. 2024), as our primary data and obtained it from the Dryad link provided from the previous publication (URL: https://datadryad.org/stash/dataset/doi:10.5061/dryad.w3r2280z5). The data contained information for 647 individuals, including 243 females, 130 males and 274 sex‐unknown individuals from 29 locations across Europe, and 483,888 genomic variants. Detailed metadata for the samples, including sequencing methods and population descriptions, are available from the original publications (Spurgin et al. 2024; Stonehouse et al. 2024). In this study, for most of our analysis, we focused on the genomic variant data from six European populations: Harjavalta (Finland), Montpellier (France), Pilis Mountains (Hungary), Seewiesen (Germany), Velky Kosir (Czechia), Wytham (UK).

### 7.2 Data Processing and Sex Identification

We first used PLINK v1.90b6.21 (Purcell et al. 2007) to filter the variants from the downloaded data by setting the parameters: ‐‐geno 0.8, ‐‐maf 0.01. We estimated the inbreeding coefficient (F) on the sex chromosome for each individual using PLINK's ‐‐check‐sex, where

$$F=\frac{\text{ObsHom}-\text{ExpHom}}{\text{ExpHom}}$$

which in our ZZ/ZW system yields F≈0 for males (ZZ) and F≈1 for females (ZW). Visualisation and quality assessment of sex‐specific classifications were performed using a customised R script with the ggplot2 package v3.5.1 (Wickham 2016). We employed the parameters ‐‐maf 0.01, ‐‐geno 0.8, ‐‐not‐chr 35, ‐‐rel‐cutoff 0.25 in subsequent analyses, consistent with the parameter values from Stonehouse et al. (2024), to filter out variants with less variation, missing data and closely related individuals.

### 7.3 Population Selection and Sampling

To minimise biases introduced by uneven sampling across populations, we selected only those populations with at least nine male and nine female individuals. Populations with fewer individuals were excluded from further analysis. Additionally, the Corsica population was excluded due to its high level of local differentiation, likely influenced by island‐specific evolutionary pressures (Spurgin et al. 2024).

### 7.4 Genomic Analyses

#### 7.4.1 Genome‐Wide Scans for Differentiation Between Males and Females

We conducted genome‐wide scans to identify sex‐specific differentiation (Weir and Cockerham 1984) across the six focal populations, each comprising nine randomly sampled males and nine randomly sampled females, using vcftools v0.1.16 (Danecek et al. 2011) with parameters ‐‐*F* _*ST*_ ‐window‐size 1 and ‐‐*F* _*ST*_ ‐window‐step 1. We calculated measures of genetic differentiation, *F*_*ST*_, at individual SNPs. We also grouped the focal locations to two subgroups for better visualisation: (1) type 1, which included Montpellier (France) Pilis Mountains (Hungary) and Velky Kosir (Czechia); (2) type 2, which included Harjavalta (Finland), Seewiesen (Germany) and Wytham (UK). Within each subgroup, we calculated *F*_*ST*_ values across chromosome five between locations and sexes with the setting mentioned in the previous paragraph.

#### 7.4.2 Impact of Sex Ratio on Differentiation

To evaluate the impact of sex ratio on genomic differentiation, we performed subsampling experiments. Subsets with varying male‐to‐female ratios (0:12, 3:9, 6:6, 9:3 and 12:0) were generated for populations from Wytham (UK) and Harjavalta (Finland) for both of which we had access to 12 females and 12 males. *F*_*ST*_ values were calculated for each subset, and patterns on chromosome 5 were visualised to assess how sex ratio influences observed differentiation.

#### 7.4.3 Whole Genome Resequencing Data

Paired‐end Illumina reads were downloaded in FASTQ format from the NCBI Sequence Read Archive (SRA) for one male and one female individual sampled from the Wytham (UK) population, respectively, using the identification number SRR2961753 (male) and SRR2961756 (female) (Laine et al. 2016). The reads were initially assessed for overall quality using FastQC v0.12.1, and low‐quality bases and adapter sequences were trimmed using Trimmomatic v0.39 (Bolger et al. 2014). The great tit reference genome (GCF_001522545.3) was obtained from NCBI. The trimmed reads were then aligned to the reference genome using the BWA‐MEM2 v2.2.1 algorithm (Li 2013) with default parameters. The SAM files were then converted to sorted BAM files and indexed using SAMtools v1.2 (Li et al. 2009). Duplicated reads in the resulting BAM files were marked using MarkDuplicatesSpark implemented in GATK v4.5.0 (Van der Auwera and O'Connor 2020). These final BAM files served as the basis for subsequent analyses, including variant visualisation with IGV v2.17.4 (Robinson et al. 2011).

### 7.5 Software and Statistical Analyses

All analyses were conducted using R v4.3.1 (R Core Team 2021), Python v3.12.2 (Van Rossum and Drake 2009), PLINK v1.90b6.21 (Purcell et al. 2007) and vcftools v0.1.16 (Danecek et al. 2011). Statistical tests and visualisations were performed in R using packages such as ggplot2 (Wickham 2016), dplyr (Wickham et al. 2023), tidyr (Wickham et al. 2024), patchwork (Pedersen 2024), stringr (Wickham 2023b) and forcats (Wickham 2023a). Scripts used for data processing and analysis are available at https://github.com/chnyuch/pma_hapmap_sex.

## Author Contributions

Toni I. Gossmann designed and supervised the study. Yu‐Chi Chen carried out analysis and provided most graphical output; all authors contributed to analysis. Toni I. Gossmann, Yu‐Chi Chen and Nikolas Vellnow wrote the manuscript. All authors contributed to writing.

## Ethics Statement

This study exclusively used publicly available data. No new samples were collected, and ethical approval was not required.

## Conflicts of Interest

The authors declare no conflicts of interest.

## Supporting information


**Data S1:** mec70061‐sup‐0001‐supinfo.pdf.

**Table S1:** Sample information and inferred sex of 647 great tit ( *Parus major* ) individuals from 29 European populations included in the HapMap data set (Spurgin et al. 2024; Stonehouse et al. 2024). Sex was determined using heterozygosity on the Z chromosome (PLINK inbreeding coefficient *F*, see Methods). Columns indicate population name, geographic coordinates, total number of individuals sampled, number of males (ZZ), females (ZW) and individuals with undetermined sex, along with metadata on sample origin. These data were used to assess sex‐specific genomic differentiation and to construct balanced subsets for downstream population genomic analyses.

## Data Availability

The data supporting the findings of this study are publicly available through the Dryad Digital Repository (DOI: https://datadryad.org/stash/dataset/doi:10.5061/dryad.w3r2280z5) and the NCBI Sequence Read Archive (SRA) for raw sequencing reads from individual great tits under accession numbers SRR2961753 and SRR2961756. The scripts used for data analysis are available on GitHub at https://github.com/chnyuch/pma_hapmap_sex. Detailed metadata, including population descriptions and sequencing methods, are included in the associated publications.

## References

[mec70061-bib-0001] Bailey, S. , J. Guhlin , D. S. Senanayake , et al. 2023. “Assembly of Female and Male Hihi Genomes (Stitchbird; *Notiomystis Cincta*) Enables Characterization of the W Chromosome and Resources for Conservation Genomics.” Molecular Ecology Resources 25, no. 5: e13823. 10.1111/1755-0998.13823. 37332137 PMC12142724

[mec70061-bib-0002] Benson, D. A. , M. Cavanaugh , K. Clark , et al. 2012. “GenBank.” Nucleic Acids Research 41, no. D1: D36–D42. 10.1093/nar/gks1195. 23193287 PMC3531190

[mec70061-bib-0003] Bolger, A. M. , M. Lohse , and B. Usadel . 2014. “Trimmomatic: A Flexible Trimmer for Illumina Sequence Data.” Bioinformatics 30, no. 15: 2114–2120. 10.1093/bioinformatics/btu170. 24695404 PMC4103590

[mec70061-bib-0004] Burri, R. , A. Nater , T. Kawakami , et al. 2015. “Linked Selection and Recombination Rate Variation Drive the Evolution of the Genomic Landscape of Differentiation Across the Speciation Continuum of Ficedula Flycatchers.” Genome Research 25, no. 11: 1656–1665. 10.1101/gr.196485.115. 26355005 PMC4617962

[mec70061-bib-0005] Camacho, C. , G. Coulouris , V. Avagyan , et al. 2009. “BLAST+: Architecture and Applications.” BMC Bioinformatics 10, no. 1: 421. 10.1186/1471-2105-10-421. 20003500 PMC2803857

[mec70061-bib-0006] Campagna, L. , and D. P. L. Toews . 2022. “The Genomics of Adaptation in Birds.” Current Biology 32, no. 20: R1173–R1186. 10.1016/j.cub.2022.07.076. 36283387

[mec70061-bib-0007] Chen, Y.‐C. , D. L. J. Vendrami , M. L. Huber , et al. 2025. “Diverse Evolutionary Trajectories of Mitocoding DNA in Mammalian and Avian Nuclear Genomes.” Genome Research 35, no. 6: 1313–1324. 10.1101/gr.279428.124. 40164502 PMC12129016

[mec70061-bib-0008] Cheng, C. , and M. Kirkpatrick . 2016. “Sex‐Specific Selection and Sex‐Biased Gene Expression in Humans and Flies.” PLoS Genetics 12, no. 9: e1006170. 10.1371/journal.pgen.1006170. 27658217 PMC5033347

[mec70061-bib-0009] Cheng, J. Y. , A. J. Stern , F. Racimo , and R. Nielsen . 2021. “Detecting Selection in Multiple Populations by Modeling Ancestral Admixture Components.” Molecular Biology and Evolution 39, no. 1: msab294. 10.1093/molbev/msab294. PMC8763095 34626111

[mec70061-bib-0010] Corcoran, P. , T. I. Gossmann , H. J. Barton , The Great Tit HapMap Consortium , J. Slate , and K. Zeng . 2017. “Determinants of the Efficacy of Natural Selection on Coding and Noncoding Variability in Two Passerine Species.” Genome Biology and Evolution 9, no. 11: 2987–3007. 10.1093/gbe/evx213. 29045655 PMC5714183

[mec70061-bib-0011] Danecek, P. , A. Auton , G. Abecasis , et al. 2011. “The Variant Call Format and VCFtools.” Bioinformatics 27, no. 15: 2156–2158 1367–4803. 10.1093/bioinformatics/btr330. 21653522 PMC3137218

[mec70061-bib-0012] Delaitre, S. , K. van Oers , M. E. Visser , and S. P. Caro . 2023. “Female Great Tits ( *Parus Major* ) Reproduce Earlier When Paired With a Male They Prefer.” Ethology 129, no. 9: 461–471. 10.1111/eth.13381.

[mec70061-bib-0013] Delmore, K. E. , B. M. van Doren , K. Ullrich , T. Curk , H. P. van der Jeugd , and M. Liedvogel . 2023. “Structural Genomic Variation and Migratory Behavior in a Wild Songbird.” Evolution Letters 7, no. 6: 401–412. 10.1093/evlett/qrad040. 38045725 PMC10693001

[mec70061-bib-0014] Ellegren, H. , and N. Galtier . 2016. “Determinants of Genetic Diversity.” Nature Reviews Genetics 17, no. 7: 422–433. 10.1038/nrg.2016.58. 27265362

[mec70061-bib-0015] Flintham, E. , V. Savolainen , S. P. Otto , M. Reuter , and C. Mullon . 2024. “The Maintenance of Genetic Polymorphism Underlying Sexually Antagonistic Traits.” Evolution Letters 9, no. 2: 259–272. 10.1093/evlett/qrae059. 40191410 PMC11968185

[mec70061-bib-0016] Foll, M. , and O. Gaggiotti . 2008. “A Genome‐Scan Method to Identify Selected Loci Appropriate for Both Dominant and Codominant Markers: A Bayesian Perspective.” Genetics 180, no. 2: 977–993. 10.1534/genetics.108.092221. 18780740 PMC2567396

[mec70061-bib-0017] Frankham, R. , J. D. Ballou , and D. A. Briscoe . 2010. Introduction to Conservation Genetics. Cambridge University Press. 10.1017/cbo9780511809002.

[mec70061-bib-0018] Gong, J. , B. Li , J. Zhao , et al. 2022. “Sex‐Specific Genomic Region Identification and Molecular Sex Marker Development of Rock Bream (*Oplegnathus fasciatus*).” Marine Biotechnology 24, no. 1: 163–173. 10.1007/s10126-022-10095-2. 35122574

[mec70061-bib-0019] Gossmann, T. I. , B. H. Song , A. J. Windsor , et al. 2010. “Genome Wide Analyses Reveal Little Evidence for Adaptive Evolution in Many Plant Species.” Molecular Biology and Evolution 27, no. 8: 1822–1832. 10.1093/molbev/msq079. 20299543 PMC2915642

[mec70061-bib-0020] Gossmann, T. I. , A. W. Santure , B. C. Sheldon , J. Slate , and K. Zeng . 2014. “Highly Variable Recombinational Landscape Modulates Efficacy of Natural Selection in Birds.” Genome Biology and Evolution 6, no. 8: 2061–2075. 10.1093/gbe/evu157. 25062920 PMC4231635

[mec70061-bib-0021] Gossmann, T. I. , A. Shanmugasundram , S. Börno , et al. 2019. “Ice‐Age Climate Adaptations Trap the Alpine Marmot in a State of Low Genetic Diversity.” Current Biology 29, no. 10: 1712–1720.e7. 10.1016/j.cub.2019.04.020. 31080084 PMC6538971

[mec70061-bib-0022] Haller, B. C. , and P. W. Messer . 2023. “SLiM 4: Multispecies Eco‐Evolutionary Modeling.” American Naturalist 201, no. 5: E127–E139. 10.1086/723601. PMC10793872 37130229

[mec70061-bib-0023] He, Q. , K. Ye , W. Han , et al. 2022. “Mapping Sex‐Determination Region and Screening DNA Markers for Genetic Sex Identification in Largemouth Bass (*Micropterus salmoides*).” Aquaculture 559: 738450. 10.1016/j.aquaculture.2022.738450. https://www.sciencedirect.com/science/article/pii/S004484862200566X.

[mec70061-bib-0024] Hoban, S. , J. L. Kelley , K. E. Lotterhos , et al. 2016. “Finding the Genomic Basis of Local Adaptation: Pitfalls, Practical Solutions, and Future Directions.” American Naturalist 188, no. 4: 379–397. 10.1086/688018. PMC5457800 27622873

[mec70061-bib-0025] Hoffman, J. I. , D. L. J. Vendrami , K. Hench , et al. 2024. “Genomic and Fitness Consequences of a Near‐Extinction Event in the Northern Elephant Seal.” Nature Ecology & Evolution 8, no. 12: 2309–2324. 10.1038/s41559-024-02533-2. 39333394 PMC11618080

[mec70061-bib-0026] Hoffmann, A. A. , and C. M. Sgrò . 2011. “Climate Change and Evolutionary Adaptation.” Nature 470, no. 7335: 479–485. 10.1038/nature09670. 21350480

[mec70061-bib-0027] Huang, H. , and D. L. Rabosky . 2015. “Sex‐Linked Genomic Variation and Its Relationship to Avian Plumage Dichromatism and Sexual Selection.” BMC Evolutionary Biology 15, no. 1: 199. 10.1186/s12862-015-0480-4. 26377432 PMC4574164

[mec70061-bib-0028] Jump, A. S. , and J. Peñuelas . 2005. “Running to Stand Still: Adaptation and the Response of Plants to Rapid Climate Change.” Ecology Letters 8, no. 9: 1010–1020. 10.1111/j.1461-0248.2005.00796.x. 34517682

[mec70061-bib-0029] Kawecki, T. J. , and D. Ebert . 2004. “Conceptual Issues in Local Adaptation.” Ecology Letters 7, no. 12: 1225–1241. 10.1111/j.1461-0248.2004.00684.x.

[mec70061-bib-0030] Laine, V. N. , T. I. Gossmann , K. M. Schachtschneider , et al. 2016. “Evolutionary Signals of Selection on Cognition From the Great Tit Genome and Methylome.” Nature Communications 7, no. 1: 10474. 10.1038/ncomms10474. PMC4737754 26805030

[mec70061-bib-0031] Lasne, C. , C. M. Sgrò , and T. Connallon . 2017. “The Relative Contributions of the *X* Chromosome and Autosomes to Local Adaptation.” Genetics 205, no. 3: 1285–1304. 10.1534/genetics.116.194670. 28064164 PMC5340339

[mec70061-bib-0032] Li, H. 2013. “Aligning Sequence Reads, Clone Sequences and Assembly Contigs With BWA‐MEM.” 10.48550/ARXIV.1303.3997.

[mec70061-bib-0033] Li, H. , B. Handsaker , A. Wysoker , et al. 2009. “The Sequence Alignment/Map Format and SAMtools.” Bioinformatics 25, no. 16: 2078–2079. 10.1093/bioinformatics/btp352. 19505943 PMC2723002

[mec70061-bib-0034] Li, M.‐H. , and J. Merilä . 2010. “Sex‐Specific Population Structure, Natural Selection, and Linkage Disequilibrium in a Wild Bird Population as Revealed by Genome‐Wide Microsatellite Analyses.” BMC Evolutionary Biology 10, no. 1: 66. 10.1186/1471-2148-10-66. 20211004 PMC2846931

[mec70061-bib-0035] Lindner, M. , I. Verhagen , A. C. Mateman , K. van Oers , V. N. Laine , and M. E. Visser . 2024. “Genetic and Epigenetic Differentiation in Response to Genomic Selection for Avian Lay Date.” Evolutionary Applications 17, no. 7: e13703. 10.1111/eva.13703. 38948539 PMC11211926

[mec70061-bib-0036] Luikart, G. , P. R. England , D. Tallmon , S. Jordan , and P. Taberlet . 2003. “The Power and Promise of Population Genomics: From Genotyping to Genome Typing.” Nature Reviews Genetics 4, no. 12: 981–994. 10.1038/nrg1226. 14631358

[mec70061-bib-0037] Lyam, P. T. , J. Duque‐Lazo , F. Hauenschild , et al. 2022. “Climate Change Will Disproportionally Affect the Most Genetically Diverse Lineages of a Widespread African Tree Species.” Scientific Reports 12, no. 1: 7035. 10.1038/s41598-022-11182-z. 35488120 PMC9054768

[mec70061-bib-0038] Makova, K. D. , B. D. Pickett , R. S. Harris , et al. 2024. “The Complete Sequence and Comparative Analysis of Ape Sex Chromosomes.” Nature 630, no. 8016: 401–411. 10.1038/s41586-024-07473-2. 38811727 PMC11168930

[mec70061-bib-0039] Moritz, C. 1994. “Defining ‘Evolutionarily Significant Units’ for Conservation.” Trends in Ecology & Evolution 9, no. 10: 373–375. 10.1016/0169-5347(94)90057-4. 21236896

[mec70061-bib-0040] Nazareno, A. G. , J. B. Bemmels , C. W. Dick , and L. G. Lohmann . 2017. “Minimum Sample Sizes for Population Genomics: An Empirical Study From an Amazonian Plant Species.” Molecular Ecology Resources 17, no. 6: 1136–1147. 10.1111/1755-0998.12654. 28078808

[mec70061-bib-0041] van Oers, K. , K. van den Heuvel , and B. Sepers . 2023. “The Epigenetics of Animal Personality.” Neuroscience and Biobehavioral Reviews 150: 105194. 10.1016/j.neubiorev.2023.105194. 37094740

[mec70061-bib-0042] Ord, J. , T. I. Gossmann , and I. Adrian‐Kalchhauser . 2023. “High Nucleotide Diversity Accompanies Differential DNA Methylation in Naturally Diverging Populations.” Molecular Biology and Evolution 40, no. 4: msad068. 10.1093/molbev/msad068. 36947101 PMC10139703

[mec70061-bib-0043] Pearman, W. S. , L. Urban , and A. Alexander . 2022. “Commonly Used Hardy–Weinberg Equilibrium Filtering Schemes Impact Population Structure Inferences Using RADseq Data.” Molecular Ecology Resources 22, no. 7: 2599–2613. 10.1111/1755-0998.13646. 35593534 PMC9541430

[mec70061-bib-0044] Pedersen, T. L. 2024. “patchwork: The Composer of Plots.” R package version 1.3.0.9000. https://github.com/thomasp85/patchwork; https://patchwork.data‐imaginist.com.

[mec70061-bib-0045] Popovic, D. , and D. B. Lowry . 2020. “Contrasting Environmental Factors Drive Local Adaptation at Opposite Ends of an Environmental Gradient in the Yellow Monkeyflower ( *Mimulus Guttatus* ).” American Journal of Botany 107, no. 2: 298–307. 10.1002/ajb2.1419. 31989586

[mec70061-bib-0046] Purcell, S. , B. Neale , K. Todd‐Brown , et al. 2007. “PLINK: A Tool Set for Whole‐Genome Association and Population‐Based Linkage Analyses.” American Journal of Human Genetics 81, no. 3: 559–575. 10.1086/519795. https://www.sciencedirect.com/science/article/pii/S0002929707613524. 17701901 PMC1950838

[mec70061-bib-0047] R Core Team . 2021. R: A Language and Environment for Statistical Computing. R Foundation for Statistical Computing. https://www.R‐project.org/.

[mec70061-bib-0048] Robinson, J. T. , H. Thorvaldsdóttir , W. Winckler , et al. 2011. “Integrative Genomics Viewer.” Nature Biotechnology 29, no. 1: 24–26. 10.1038/nbt.1754. PMC3346182 21221095

[mec70061-bib-0049] Rodrigues, N. , and C. Dufresnes . 2017. “Using Conventional F‐Statistics to Study Unconventional Sex‐Chromosome Differentiation.” PeerJ 5: e3207. 10.7717/peerj.3207. 28462023 PMC5410149

[mec70061-bib-0050] Rogers, T. F. , D. H. Palmer , and A. E. Wright . 2020. “Sex‐Specific Selection Drives the Evolution of Alternative Splicing in Birds.” Molecular Biology and Evolution 38, no. 2: 519–530. 10.1093/molbev/msaa242. PMC7826194 32977339

[mec70061-bib-0051] Romiguier, J. , P. Gayral , M. Ballenghien , et al. 2014. “Comparative Population Genomics in Animals Uncovers the Determinants of Genetic Diversity.” Nature 515, no. 7526: 261–263. 10.1038/nature13685. 25141177

[mec70061-bib-0052] Ruzicka, F. , L. Dutoit , P. Czuppon , et al. 2020. “The Search for Sexually Antagonistic Genes: Practical Insights From Studies of Local Adaptation and Statistical Genomics.” Evolution Letters 4, no. 5: 398–415. 10.1002/evl3.192. 33014417 PMC7523564

[mec70061-bib-0053] Santure, A. W. , and D. Garant . 2018. “Wild GWAS—Association Mapping in Natural Populations.” Molecular Ecology Resources 18, no. 4: 729–738. 10.1111/1755-0998.12901. 29782705

[mec70061-bib-0054] Savolainen, O. , M. Lascoux , and J. Merilä . 2013. “Ecological Genomics of Local Adaptation.” Nature Reviews Genetics 14, no. 11: 807–820. 10.1038/nrg3522. 24136507

[mec70061-bib-0055] Spurgin, L. G. , M. Bosse , F. Adriaensen , et al. 2024. “The Great Tit HapMap Project: A Continental‐Scale Analysis of Genomic Variation in a Songbird.” Molecular Ecology Resources 24, no. 5: e13969. 10.1111/1755-0998.13969. 38747336

[mec70061-bib-0056] Stonehouse, J. C. , L. G. Spurgin , V. N. Laine , et al. 2024. “The Genomics of Adaptation to Climate in European Great Tit ( *Parus Major* ) Populations.” Evolution Letters 8, no. 1: 18–28. 10.1093/evlett/qrad043. 38370545 PMC10872194

[mec70061-bib-0057] Thorburn, D.‐M. J. , K. Sagonas , M. Binzer‐Panchal , et al. 2023. “Origin Matters: Using a Local Reference Genome Improves Measures in Population Genomics.” Molecular Ecology Resources 23, no. 7: 1706–1723. 10.1111/1755-0998.13838. 37489282

[mec70061-bib-0058] van den Heuvel, K. , J. L. Quinn , A. Kotrschal , and K. van Oers . 2023. “Artificial Selection for Reversal Learning Reveals Limited Repeatability and no Heritability of Cognitive Flexibility in Great Tits (*Parus major*).” Proceedings of the Royal Society B: Biological Sciences 290: 2003. 10.1098/rspb.2023.1067. PMC10354490 37464752

[mec70061-bib-0059] Van der Auwera, G. A. , and B. D. O'Connor . 2020. Genomics in the Cloud: Using Docker, GATK, and WDL in Terra. 1st ed. O'Reilly Media.

[mec70061-bib-0060] Van Rossum, G. , and F. L. Drake . 2009. Python 3 Reference Manual. CreateSpace.

[mec70061-bib-0061] Weir, B. S. , and C. C. Cockerham . 1984. “Estimating F‐Statistics for the Analysis of Population Structure.” Evolution 38, no. 6: 1358. 10.2307/2408641. 28563791

[mec70061-bib-0062] Wickham, H. 2016. ggplot2: Elegant Graphics for Data Analysis. Springer‐Verlag. https://ggplot2.tidyverse.org.

[mec70061-bib-0063] Wickham, H. 2023a. “forcats: Tools for Working With Categorical Variables (Factors).” R package version 1.0.0. https://github.com/tidyverse/forcats; https://forcats.tidyverse.org/.

[mec70061-bib-0064] Wickham, H. 2023b. “stringr: Simple, Consistent Wrappers for Common String Operations.” R package version 1.5.1. https://github.com/tidyverse/stringr; https://stringr.tidyverse.org.

[mec70061-bib-0065] Wickham, H. , R. François , L. Henry , K. Müller , and D. Vaughan . 2023. “dplyr: A Grammar of Data Manipulation.” R package version 1.1.4. https://github.com/tidyverse/dplyr; https://dplyr.tidyverse.org.

[mec70061-bib-0066] Wickham, H. , D. Vaughan , and M. Girlich . 2024. “tidyr: Tidy Messy Data.” R package version 1.3.1. https://github.com/tidyverse/tidyr; https://tidyr.tidyverse.org.

[mec70061-bib-0067] Ye, Z. , W. Wei , M. E. Pfrender , and M. Lynch . 2023. “Evolutionary Insights From a Large‐Scale Survey of Population‐Genomic Variation.” Molecular Biology and Evolution 40, no. 11: msad233. 10.1093/molbev/msad233. 37863047 PMC10630549

[mec70061-bib-0068] Yi, X. , Y. Liang , E. Huerta‐Sanchez , et al. 2010. “Sequencing of 50 Human Exomes Reveals Adaptation to High Altitude.” Science 329, no. 5987: 75–78. 10.1126/science.1190371. 20595611 PMC3711608

