# Supplementary material for: Opinion: Why Sex‐Based Genomic Differentiation Should Not Be Overlooked in Population Genetics
Source: Mol Ecol. 2025 Aug 7;34(18):e70061. doi: 10.1111/mec.70061 (PMC12421491; doi:10.1111/mec.70061)
Supplement: Supplementary file 1 — Data S1: mec70061‐sup‐0001‐supinfo.pdf. [file MEC-34-e70061-s002.pdf]

Supplementary Information

Description	Scientific Name	Common Name	Taxid	Max Score	Total Score	Query cover	E Value	Per. Ident	Acc. Len	Accession
Parus major isolate Abel chromosome 5, Parus_major1.1	Parus major	Great Tit	9157	126	126	100%	5e-26	98.59	61875929	NC_031774.1
Pseudopodoces humilis unplaced genomic scaffold, PseHum1.0...	Pseudopodoce...	Tibetan grou...	181119	126	126	96%	5e-26	100.00	101331	NW_005087830.1
Poecile atricapillus isolate bPoeAtr1 chromosome W, bPoeAtr1.hap1	Poecile atri...	Black-capped...	48891	121	1519	100%	2e-24	98.53	107919339	NC_081288.1
Poecile atricapillus isolate bPoeAtr1 chromosome Z, bPoeAtr1.hap1	Poecile atri...	Black-capped...	48891	121	2426	100%	2e-24	97.18	146584261	NC_081289.1
Pseudopodoces humilis unplaced genomic scaffold, PseHum1.0...	Pseudopodoce...	Tibetan grou...	181119	121	121	96%	2e-24	98.53	859909	NW_005087679.1
Poecile atricapillus isolate bPoeAtr1 chromosome 32,...	Poecile atri...	Black-capped...	48891	115	935	100%	1e-22	95.77	4032282	NC_081280.1
Poecile atricapillus isolate bPoeAtr1 chromosome 26,...	Poecile atri...	Black-capped...	48891	110	979	100%	5e-21	94.37	7054485	NC_081274.1
Parus major isolate Abel chromosome Z, Parus_major1.1	Parus major	Great Tit	9157	110	110	96%	5e-21	95.59	74514349	NC_031799.1
Anomalospiza imberbis isolate Cuckoo-Finch-1a 21T00152...	Anomalospiza...	NA	187417	78.7	1560	100%	1e-11	86.96	7764622	NC_089720.1
Chamaea fasciata isolate MVZ Bird 193981 ecotype frontalis...	Chamaea fasc...	NA	190680	73.1	73.1	93%	7e-10	86.36	84192302	NW_027094089.1
Melozone crissalis strain CALT ecotype California unplaced...	Melozone cri...	California t...	40204	73.1	124	93%	7e-10	86.36	4882851	NW_026535882.1
Ammospiza nelsoni isolate bAmmNel1 chromosome W, bAmmNel1.pri	Ammospiza ne...	NA	2857394	73.1	348	93%	7e-10	86.36	21546180	NC_080668.1
Hirundo rustica isolate bHirRus1 chromosome W, bHirRus1.pri.v3	Hirundo rustica	Barn swallow	43150	73.1	4077	93%	7e-10	92.16	31704074	NC_053487.1
Sylvia atricapilla isolate bSylAtr1 chromosome W, bSylAtr1.pri	Sylvia atric...	blackcap	48155	73.1	310	93%	7e-10	86.36	21688951	NC_089173.1
Corvus moneduloides isolate bCorMon1 chromosome W, bCorMon1.pri	Corvus moned...	New Caledoni...	1196302	73.1	1862	93%	7e-10	92.16	21203907	NC_045510.1
Corvus brachyrhynchos isolate BGI_N302 unplaced genomic...	Corvus brach...	American crow	85066	73.1	73.1	72%	7e-10	92.16	8233	NW_008236242.1
Haemorhous mexicanus isolate bHaeMex1 chromosome W, bHaeMex1.pri	Haemorhous m...	house finch	30427	69.4	307	93%	8e-09	95.35	9455355	NC_082380.1
Taeniopygia guttata chromosome W, bTaeGut7.mat	Taeniopygia ...	zebra finch	59729	67.6	559	93%	3e-08	97.44	33777440	NC_133064.1
Aphelocoma coerulescens isolate FSJ_1873_10779 chromosome W...	Aphelocoma c...	scrub jay	39617	67.6	1352	93%	3e-08	90.20	20979278	NW_027184080.1
Aphelocoma coerulescens isolate FSJ_1873_10779 chromosome W...	Aphelocoma c...	scrub jay	39617	67.6	191	72%	3e-08	90.20	9289604	NW_027184081.1
Aphelocoma coerulescens isolate FSJ_1873_10779 chromosome W...	Aphelocoma c...	scrub jay	39617	67.6	315	93%	3e-08	84.85	4676095	NW_027184082.1
Aphelocoma coerulescens isolate FSJ_1873_10779 chromosome W...	Aphelocoma c...	scrub jay	39617	67.6	191	72%	3e-08	90.20	3429207	NW_027184083.1
Aphelocoma coerulescens isolate FSJ_1873_10779 chromosome W...	Aphelocoma c...	scrub jay	39617	67.6	248	72%	3e-08	90.20	3082033	NW_027184084.1
Melospiza melodia melodia isolate bMelMel2 chromosome W,...	Melospiza me...	NA	1914991	67.6	519	93%	3e-08	84.85	59460375	NC_086225.1
Anomalospiza imberbis isolate Cuckoo-Finch-1a 21T00152...	Anomalospiza...	NA	187417	67.6	124	93%	3e-08	84.85	312075	NW_027099294.1
Zonotrichia leucophrys gambelii isolate GWCS_2022_RI unplaced...	Zonotrichia ...	NA	257770	67.6	67.6	93%	3e-08	84.85	1714398	NW_026992238.1
Melospiza georgiana isolate bMelGeo1 chromosome W, bMelGeo1.pri	Melospiza ge...	NA	44398	67.6	380	93%	3e-08	84.85	34706425	NC_080464.1
Vidua chalybeata isolate OUT-0048 chromosome W unlocalized...	Vidua chalyb...	NA	81927	67.6	226	73%	3e-08	95.24	2604421	NW_026530320.1
Vidua chalybeata isolate OUT-0048 chromosome W unlocalized...	Vidua chalyb...	NA	81927	67.6	124	93%	3e-08	84.85	984458	NW_026530343.1
Vidua macroura isolate BioBank_ID:100142 chromosome W,...	Vidua macroura	Pin-tailed w...	187451	67.6	227	93%	3e-08	84.85	7618882	NC_071610.1
Prinia subflava isolate CZ2003 ecotype Zambia chromosome W,...	Prinia subflava	tawny-flanke...	208062	67.6	191	59%	3e-08	97.44	10855263	NC_086282.1
Corvus hawaiiensis isolate bCorHaw1 chromosome 3,...	Corvus hawai...	hawaiian crow	134902	67.6	67.6	72%	3e-08	90.20	121534940	NC_063215.1
Corvus hawaiiensis isolate bCorHaw1 chromosome W,...	Corvus hawai...	hawaiian crow	134902	67.6	552	93%	3e-08	90.20	6742290	NC_063254.1
Corvus kubaryi isolate FWS band 111438036 unplaced genomic...	Corvus kubaryi	Mariana crow	68294	67.6	67.6	72%	3e-08	90.20	5721746	NW_024582058.1
Pyrgilauda ruficollis isolate IOZ18807 unplaced genomic...	Pyrgilauda r...	rufous-necke...	221976	67.6	67.6	93%	3e-08	85.07	32520	NW_024527111.1
Corvus moneduloides isolate bCorMon1 chromosome 3, bCorMon1.pri	Corvus moned...	New Caledoni...	1196302	67.6	67.6	72%	3e-08	90.20	122098567	NC_045478.1
Corvus moneduloides isolate bCorMon1 chromosome 30, bCorMon1.pri	Corvus moned...	New Caledoni...	1196302	67.6	67.6	72%	3e-08	90.20	2735592	NC_045505.1
Geospiza fortis unplaced genomic scaffold, GeoFor_1.0 scaffold493	Geospiza fortis	medium groun...	48883	67.6	67.6	55%	3e-08	97.44	23353	NW_005055100.1
Aphelocoma coerulescens isolate FSJ_1873_10779 chromosome 4A,...	Aphelocoma c...	scrub jay	39617	63.9	116	70%	4e-07	90.00	21932799	NC_091018.1
Passer domesticus isolate bPasDom1 chromosome W, bPasDom1.hap1	Passer domes...	house sparrow	48849	63.9	393	93%	4e-07	93.02	47418805	NC_087511.1
Agelaius tricolor isolate 1412-34295 unplaced genomic scaffold...	Agelaius tri...	tricolored b...	9191	63.9	63.9	61%	4e-07	93.02	2357813	NW_027269316.1
Serinus canaria isolate serCan28SL12 chromosome W, serCan2020	Serinus canaria	Common canary	9135	63.9	478	93%	4e-07	88.46	18818188	NC_066342.1
Prinia subflava isolate CZ2003 ecotype Zambia chromosome W...	Prinia subflava	tawny-flanke...	208062	63.9	63.9	61%	4e-07	93.02	2886503	NW_026960611.1
Agelaius phoeniceus isolate BioBank_ID:100140 chromosome W...	Agelaius pho...	red-winged b...	39638	63.9	63.9	73%	4e-07	88.46	1602209	NW_026530958.1
Catharus ustulatus isolate bCatUst1 chromosome W, bCatUst1.pri.v2	Catharus ust...	Swainson's t...	91951	63.9	335	93%	4e-07	93.02	12285394	NC_046261.2
Geospiza fortis unplaced genomic scaffold, GeoFor_1.0...	Geospiza fortis	medium groun...	48883	63.9	63.9	61%	4e-07	93.02	10567	NW_005055439.1
Geospiza fortis unplaced genomic scaffold, GeoFor_1.0...	Geospiza fortis	medium groun...	48883	63.9	63.9	61%	4e-07	93.02	463	NW_005071157.1
Molothrus aeneus isolate 106 chromosome W, BPBGC_Maene_1.0	Molothrus ae...	bronzed cowbird	84833	62.1	262	93%	1e-06	83.33	12863401	NC_089679.1
Anomalospiza imberbis isolate Cuckoo-Finch-1a 21T00152...	Anomalospiza...	NA	187417	62.1	708	93%	1e-06	83.33	7269374	NW_027099315.1
Anomalospiza imberbis isolate Cuckoo-Finch-1a 21T00152...	Anomalospiza...	NA	187417	62.1	62.1	59%	1e-06	92.86	561516	NW_027099339.1
Zonotrichia leucophrys gambelii isolate GWCS_2022_RI unplaced...	Zonotrichia ...	NA	257770	62.1	62.1	55%	1e-06	94.87	405039	NW_026992250.1
Zonotrichia leucophrys gambelii isolate GWCS_2022_RI unplaced...	Zonotrichia ...	NA	257770	62.1	109	93%	1e-06	88.24	374463	NW_026992256.1
Melozone crissalis strain CALT ecotype California unplaced...	Melozone cri...	California t...	40204	62.1	297	100%	1e-06	94.87	14284040	NW_026535813.1
Vidua chalybeata isolate OUT-0048 chromosome W unlocalized...	Vidua chalyb...	NA	81927	62.1	62.1	93%	1e-06	83.33	1426694	NW_026530338.1
Vidua chalybeata isolate OUT-0048 chromosome W unlocalized...	Vidua chalyb...	NA	81927	62.1	62.1	55%	1e-06	94.87	934863	NW_026530344.1
Vidua macroura isolate BioBank_ID:100142 chromosome W...	Vidua macroura	Pin-tailed w...	187451	62.1	107	75%	1e-06	92.86	7456899	NW_026530534.1
Vidua macroura isolate BioBank_ID:100142 chromosome W...	Vidua macroura	Pin-tailed w...	187451	62.1	124	72%	1e-06	88.24	4182901	NW_026530536.1
Vidua macroura isolate BioBank_ID:100142 chromosome W...	Vidua macroura	Pin-tailed w...	187451	62.1	62.1	59%	1e-06	92.86	2386442	NW_026530537.1
Vidua macroura isolate BioBank_ID:100142 unplaced genomic...	Vidua macroura	Pin-tailed w...	187451	62.1	62.1	72%	1e-06	88.24	526329	NW_026530546.1
Agelaius tricolor isolate 1412-34295 unplaced genomic scaffold...	Agelaius tri...	tricolored b...	9191	62.1	601	93%	1e-06	83.33	14783721	NW_027269281.1
Prinia subflava isolate CZ2003 ecotype Zambia chromosome W...	Prinia subflava	tawny-flanke...	208062	62.1	62.1	55%	1e-06	94.87	8816889	NW_026960606.1
Agelaius phoeniceus isolate BioBank_ID:100140 chromosome W,...	Agelaius pho...	red-winged b...	39638	62.1	390	93%	1e-06	83.33	9221756	NC_071709.1
Agelaius phoeniceus isolate BioBank_ID:100140 chromosome W...	Agelaius pho...	red-winged b...	39638	62.1	62.1	93%	1e-06	83.33	1453837	NW_026530959.1
Agelaius phoeniceus isolate BioBank_ID:100140 unplaced genomic...	Agelaius pho...	red-winged b...	39638	62.1	62.1	93%	1e-06	83.33	428976	NW_026530998.1
Corvus hawaiiensis isolate bCorHaw1 chromosome 13,...	Corvus hawai...	hawaiian crow	134902	62.1	62.1	72%	1e-06	88.24	22362767	NC_063225.1
Corvus kubaryi isolate FWS band 111438036 unplaced genomic...	Corvus kubaryi	Mariana crow	68294	62.1	62.1	72%	1e-06	88.24	1764201	NW_024581071.1
Pyrgilauda ruficollis isolate IOZ18807 unplaced genomic...	Pyrgilauda r...	rufous-necke...	221976	62.1	62.1	59%	1e-06	92.86	24317	NW_024528211.1
Hirundo rustica isolate bHirRus1 chromosome 1, bHirRus1.pri.v3	Hirundo rustica	Barn swallow	43150	62.1	271	72%	1e-06	88.24	156035725	NC_053450.1
Hirundo rustica isolate bHirRus1 chromosome 4, bHirRus1.pri.v3	Hirundo rustica	Barn swallow	43150	62.1	162	61%	1e-06	92.86	76187387	NC_053453.1
Molothrus ater isolate BHLD 08-10-18 breed brown headed cowbir...	Molothrus ater	NA	84834	62.1	165	93%	1e-06	92.86	11773180	NC_050510.2
Corvus moneduloides isolate bCorMon1 chromosome 13, bCorMon1.pri	Corvus moned...	New Caledoni...	1196302	62.1	62.1	72%	1e-06	88.24	20922009	NC_045488.1
Corvus brachyrhynchos isolate BGI_N302 unplaced genomic...	Corvus brach...	American crow	85066	62.1	62.1	72%	1e-06	88.24	25110	NW_008235690.1
Corvus brachyrhynchos isolate BGI_N302 unplaced genomic...	Corvus brach...	American crow	85066	62.1	62.1	72%	1e-06	88.24	68455	NW_008236785.1
Corvus brachyrhynchos isolate BGI_N302 unplaced genomic...	Corvus brach...	American crow	85066	62.1	62.1	72%	1e-06	88.24	81159	NW_008236991.1
Corvus brachyrhynchos isolate BGI_N302 unplaced genomic...	Corvus brach...	American crow	85066	62.1	62.1	72%	1e-06	88.24	25941	NW_008238657.1
Geospiza fortis unplaced genomic scaffold, GeoFor_1.0 scaffold454	Geospiza fortis	medium groun...	48883	62.1	62.1	59%	1e-06	92.86	13479	NW_005055314.1
Geospiza fortis unplaced genomic scaffold, GeoFor_1.0...	Geospiza fortis	medium groun...	48883	62.1	62.1	93%	1e-06	83.33	6413	NW_005055762.1
Anomalospiza imberbis isolate Cuckoo-Finch-1a 21T00152...	Anomalospiza...	NA	187417	60.2	60.2	58%	5e-06	92.68	72455167	NC_089685.1
Anomalospiza imberbis isolate Cuckoo-Finch-1a 21T00152...	Anomalospiza...	NA	187417	60.2	60.2	58%	5e-06	92.68	11345200	NC_089701.1
Anomalospiza imberbis isolate Cuckoo-Finch-1a 21T00152 unplace...	Anomalospiza...	NA	187417	60.2	60.2	58%	5e-06	92.68	303791	NW_027099470.1
Corvus hawaiiensis isolate bCorHaw1 chromosome 11,...	Corvus hawai...	hawaiian crow	134902	60.2	60.2	59%	5e-06	92.86	23986915	NC_063223.1
Corvus moneduloides isolate bCorMon1 chromosome 11, bCorMon1.pri	Corvus moned...	New Caledoni...	1196302	60.2	60.2	55%	5e-06	94.87	21791607	NC_045486.1
Zonotrichia leucophrys gambelii isolate GWCS_2022_RI unplaced...	Zonotrichia ...	NA	257770	58.4	58.4	61%	2e-05	90.70	118765	NW_026992344.1
Corvus brachyrhynchos isolate BGI_N302 unplaced genomic...	Corvus brach...	American crow	85066	58.4	58.4	69%	2e-05	88.00	1387	NW_008236837.1
Anomalospiza imberbis isolate Cuckoo-Finch-1a 21T00152...	Anomalospiza...	NA	187417	56.5	56.5	93%	7e-05	81.82	1244791	NC_089717.1
Chamaea fasciata isolate MVZ Bird 193981 ecotype frontalis...	Chamaea fasc...	NA	190680	56.5	56.5	93%	7e-05	81.82	74821068	NW_027094090.1
Vidua chalybeata isolate OUT-0048 chromosome W, bVidCha1 merge...	Vidua chalyb...	NA	81927	56.5	105	72%	7e-05	86.27	3371560	NC_071569.1
Vidua macroura isolate BioBank_ID:100142 chromosome W...	Vidua macroura	Pin-tailed w...	187451	56.5	107	93%	7e-05	81.82	2351913	NW_026530538.1
Vidua macroura isolate BioBank_ID:100142 chromosome W...	Vidua macroura	Pin-tailed w...	187451	56.5	56.5	93%	7e-05	82.09	2208874	NW_026530539.1
Prinia subflava isolate CZ2003 ecotype Zambia chromosome W...	Prinia subflava	tawny-flanke...	208062	56.5	56.5	93%	7e-05	81.82	1074025	NW_026960615.1
Prinia subflava isolate CZ2003 ecotype Zambia unplaced genomic...	Prinia subflava	tawny-flanke...	208062	56.5	56.5	55%	7e-05	92.31	969868	NW_026961001.1
Corvus hawaiiensis isolate bCorHaw1 chromosome 24,...	Corvus hawai...	hawaiian crow	134902	56.5	56.5	55%	7e-05	92.31	11344391	NC_063236.1
Hirundo rustica isolate bHirRus1 unplaced genomic scaffold,...	Hirundo rustica	Barn swallow	43150	56.5	56.5	55%	7e-05	92.31	327343	NW_026690714.1
Hirundo rustica isolate bHirRus1 chromosome Z, bHirRus1.pri.v3	Hirundo rustica	Barn swallow	43150	56.5	162	63%	7e-05	88.89	90132487	NC_053488.1
Catharus ustulatus isolate bCatUst1 chromosome 8, bCatUst1.pri.v2	Catharus ust...	Swainson's t...	91951	56.5	56.5	61%	7e-05	90.70	36548054	NC_046228.1
Catharus ustulatus isolate bCatUst1 chromosome 25,...	Catharus ust...	Swainson's t...	91951	56.5	56.5	61%	7e-05	90.70	8277587	NC_046245.1
Catharus ustulatus isolate bCatUst1 chromosome 30,...	Catharus ust...	Swainson's t...	91951	56.5	56.5	61%	7e-05	90.70	4023379	NC_046250.1
Corvus moneduloides isolate bCorMon1 chromosome 15, bCorMon1.pri	Corvus moned...	New Caledoni...	1196302	56.5	107	63%	7e-05	88.89	19370643	NC_045490.1
Corvus moneduloides isolate bCorMon1 chromosome 24, bCorMon1.pri	Corvus moned...	New Caledoni...	1196302	56.5	56.5	55%	7e-05	92.31	7308453	NC_045499.1
Corvus moneduloides isolate bCorMon1 chromosome 28, bCorMon1.pri	Corvus moned...	New Caledoni...	1196302	56.5	56.5	72%	7e-05	86.27	5795124	NC_045503.1

Figure S1: BLAST results of the chromosome 5 probe sequence "TTCGAATATCAGCAAACACGTGCCATAAAT-GCTACCGAAAGCTGTATATGCAAAGACACCGAGGTTCCTTT" (C allele) against available bird genome assemblies (taxid:8782) on the NCBI RefSeq Genome Database. A word size of 16 and expect size of 100 was used, including low complexity filter.

```
Database: Genome (bPoeAtr1.hap1) Poecile atricapillus bPoeAtr1.hap1 [GCF_030490865.1] chromosomes plus unplaced and unlocalized scaffolds (reference assembly in GCF_030490865.1-RS_2023_08)

Sequences producing significant alignments:
Scientific      Common                    Max    Total Query   E   Per.   Acc.
Description                                                  Name            Name             Taxid      Score  Score cover Value Ident  Len        Accession
Poecile atricapillus isolate bPoeAtr1 chromosome W, bPoeAtr1.hap1 Poecile atri... Black-capped... 48891      121    891   100%  2e-26 98.53  107919339  NC_081288.1
Poecile atricapillus isolate bPoeAtr1 chromosome Z, bPoeAtr1.hap1 Poecile atri... Black-capped... 48891      121    2211  100%  2e-26 97.18  146584261  NC_081289.1
Poecile atricapillus isolate bPoeAtr1 chromosome 32,...          Poecile atri... Black-capped... 48891      115    638   100%  9e-25 95.77  4032282    NC_081280.1
Poecile atricapillus isolate bPoeAtr1 chromosome 26,...          Poecile atri... Black-capped... 48891      110    770   100%  4e-23 94.37  7054485    NC_081274.1

Alignments:

Query        1          TTCGAATATCAGCAAACACGTGCCATAAATGCTACCGAAAGCTGTATATGCAAAGACACCGAGGTTCCTTT  71
NC_081288.1  90427982   ..G.....C.............................G................................  90428052
NC_081288.1  105845792  ..G.....C...............................T..............................  105845862
NC_081288.1  65414653   ..G.....C...G.............................................T............  65414723
NC_081288.1  85643804   ..G.....C..........A....................T..............................  85643874
NC_081288.1  66348335   ..G.....C...T......A..................G................................  66348405
NC_081288.1  106653352     .....C..............................................................  106653285
NC_081288.1  66081197      .....C.........T....................................................  66081130

NC_081289.1  70851230   ..G.....C..............................................................  70851300
NC_081289.1  71313219   ..G.....C..............................................................  71313289
NC_081289.1  71343515   ..G.....C..............................................................  71343585
NC_081289.1  71543340   ..G.....C..............................................................  71543410
NC_081289.1  72503168   ..G.....C..............................................................  72503238
NC_081289.1  72622188   ..G.....C..............................................................  72622258
NC_081289.1  71948856   ..G.....C...A..........................................................  71948926
NC_081289.1  71979191   ..G.....C...A..........................................................  71979261
NC_081289.1  72418437   ..G.....C................................................T.............  72418507
NC_081289.1  71279911   ..G.....C......................................G.........T.T...........  71279981
NC_081289.1  71572699   ..G.....C......................................G.........T.T...........  71572769
NC_081289.1  71188564      .....C..............................................................  71188497
NC_081289.1  71927953      .....C..............................................................  71927886
NC_081289.1  72529941      .....C..............................................................  72529874
NC_081289.1  71225365      .....C............................G.................................  71225298
NC_081289.1  71472799      .....C..................................A...........................  71472732
NC_081289.1  78346360      .....C.........T....................................................  78346293
NC_081289.1  71719594      .....C...G......A...................................................  71719527
NC_081289.1  70841440      .....C...........................A..............................      70841377

NC_081280.1  2119434    ..G.....C...............................T..............................  2119504
NC_081280.1  1277533    ..G.....C..........A.....G..........A...T..............................  1277603
NC_081280.1  3652098       .....C..........A....................T..............................  3652031
NC_081280.1  859757        .....C..........A.....G..............T..............................  859690
NC_081280.1  2306074       .....C..........A.....G..............T..............................  2306007
NC_081280.1  2623113       .....C..........A.....G..............T..............................  2623046

NC_081274.1  2011887    ..G.....C...........A.....................................T............  2011957
NC_081274.1  3035906    ..G.....C...........A.....................................T............  3035976
NC_081274.1  3136169    ..G.....C...........A.....................................T............  3136239
NC_081274.1  2745015       .....C...........A.....................................T............  2744948
NC_081274.1  2897229       .....C...........A.....................................T............  2897296
NC_081274.1  3631287       .....C...........A.....................................T............  3631220
NC_081274.1  4081070       .....C...........A.....................................T............  4081003
```

Figure S2: BLAST results including alignment details of the chromosome 5 probe sequence (C allele) against the *Poecile atricapillus* genome assembly. Identifiers NC_081288.1 and NC_081289.1 correspond to W and Z chromosome, respectively.

Table S2: Examples of single-locus genomic configurations causing sex-specific SNP differentiation. For illustration purposes, alleles are coded as **1** (reference), **2** (alternative), and **0** (absent). Assumes ZW sex-determination (male ZZ, female ZW). The final column gives the expected FST (or differentiation) if a set of males versus females is compared, assuming species-wide patterns and mapping ambiguity.

**Scenario**	**Description**	**Male Genotype**	**Female Genotype**	**Predicted F_{ST} (M vs F)**
PAR (pseudoautosomal region)	Shared region between Z and W chromosomes	1/1	1/2	Moderate (F_{ST} up to 0.5 possible if W allele is common; not species-wide fixed)
Z-specific SNP (non-PAR)	Present only on Z chromosome; W lacks locus	1/1	1/0 (hemizygous)	Low or zero; no true allele frequency difference
W-specific SNP	Absent in males; W carries distinct allele	0/0	0/2	High, up to 1.0 (apparent fixation of alt allele in females only)
Autosomal + W-duplication (biallelic)	Locus on autosome (1/1 in both), extra W copy in females with alt allele	1/1	1/1/2 (allele ratio ∼66:33)	Moderate; FST 0.1-0.4 possible, depending on expression/mapping of W-allele
Z-duplicated + W-variant (e.g. outside of PAR)	1/1 on Z in males; W in females carries alternative allele	1/1	1/2	Moderate to high, up to 0.5 or higher if W-allele is always alternative
Z/W multiallelic expansion	Z: allele 1; W: multiple alt copies (e.g. 2 copies of allele 2)	1/1	1/2/2/2 (allele ratio ∼25:75)	High; can approach 1.0 if most reads in females are alt
Autosomal pseudogene on W	True autosomal gene (1/1), pseudogenic copy with alt allele on W	1/1	1/1/2 or 1/2 (allele inflation in females)	Low to moderate (FST 0-0.3); may inflate female heterozygosity, but effect depends on mapping stringency
Sex-linked CNV overlap	Copy number gain (e.g. duplication) exclusive to W	1/1	1/2 or higher dosage of alt allele depending on mapping	Moderate to high; FST increases with W copy dosage
